# The Consensus 5' Splice Site Motif Inhibits mRNA Nuclear Export

**DOI:** 10.1371/journal.pone.0122743

**Published:** 2015-03-31

**Authors:** Eliza S. Lee, Abdalla Akef, Kohila Mahadevan, Alexander F. Palazzo

**Affiliations:** Department of Biochemistry, University of Toronto, 1 King’s College Circle, MSB Room 5336, Toronto, ON, M5S 1A8, Canada; National Institute of Health - National Cancer Institute, UNITED STATES

## Abstract

In eukaryotes, mRNAs are synthesized in the nucleus and then exported to the cytoplasm where they are translated into proteins. We have mapped an element, which when present in the 3’terminal exon or in an unspliced mRNA, inhibits mRNA nuclear export. This element has the same sequence as the consensus 5’splice site motif that is used to define the start of introns. Previously it was shown that when this motif is retained in the mRNA, it causes defects in 3’cleavage and polyadenylation and promotes mRNA decay. Our new data indicates that this motif also inhibits nuclear export and promotes the targeting of transcripts to nuclear speckles, foci within the nucleus which have been linked to splicing. The motif, however, does not disrupt splicing or the recruitment of UAP56 or TAP/Nxf1 to the RNA, which are normally required for nuclear export. Genome wide analysis of human mRNAs, lncRNA and eRNAs indicates that this motif is depleted from naturally intronless mRNAs and eRNAs, but less so in lncRNAs. This motif is also depleted from the beginning and ends of the 3’terminal exons of spliced mRNAs, but less so for lncRNAs. Our data suggests that the presence of the 5’splice site motif in mature RNAs promotes their nuclear retention and may help to distinguish mRNAs from misprocessed transcripts and transcriptional noise.

## Introduction

In mammalian cells, intergenic transcription accounts for a large fraction of the total nascent RNA output, approximately equal to the amount of protein-coding RNA [1,2]. The vast majority of this intergenic transcription is degraded soon after synthesis and this is reflected in the fact that at steady state levels protein coding RNA is present at levels 25–100 fold greater than intergenic RNA [1–5]. It is believed that mRNA and transcriptional noise differ by the fact that the former has particular identity features, such as splice sites, poly(A)-tails and specialized sequences [6–8]. This idea is consistent with the findings that the inclusion of spliced introns into a transcript promotes the export of the mature mRNA [9–11]. In contrast, it is believed that transcripts with aberrant features, which are not usually present in mRNAs, are retained in the nucleus and targeted for degradation [7].

We previously identified an RNA element that promotes an alternative mRNA export pathway (ALREX) [10]. This element promotes the efficient nuclear export of microinjected RNA, which was synthesized *in vitro* [10]. ALREX-promoting elements also potentiate the efficient translation of the mRNA into protein [12]. Interestingly, we found that within the context of *in vivo* transcribed RNA, the element only promoted export of certain mRNAs [13]. This result indicated that supplementary features are present within various RNA transcripts that modulate the activity of the ALREX-element. Underlying these observations, was the idea that in the absence of any sequence-based elements or splicing events, a polyadenylated RNA was not a substrate for nuclear export. This idea was supported by three observations. First, certain artificial intronless mRNAs, such as the *fushi tarazu* mini gene transcript and an intronless *β-globin* mRNA, were not exported when they lacked introns or specialized export-promoting elements [9–11]. Second, intronless RNA expressed from plasmids with strong promoters and polyadenylation signals but with random sequences, seem to be inefficiently exported and have very short half-lives [14,15]. Third, naturally intronless mRNAs appear to have specialized RNA elements that promote nuclear export [15–17]. It is however possible that these various export-deficient intronless mRNAs may contain elements that inhibit their export. This idea is supported by the fact that most intronless mRNAs are in fact efficiently exported from the nucleus [7], and that certain long noncoding RNAs are retained in the nucleus by specific motifs and once these elements are eliminated, the lncRNAs start to accumulate in the cytoplasm [18–20]. In further support of this idea, we have also recently discovered that the intronless *β-globin* mRNA contains a region that actively inhibits the export of short intronless mRNAs (A. Akef and A. Palazzo, manuscript in preparation).

Here we demonstrate that the *fushi tarazu* mini gene transcript, used by our lab in several published studies, contains an element that inhibits mRNA export. We mapped this element to the plasmid vector backbone, between the 3’end of the *ftz* insert and the 3’processing signal. This element is present in a variety of commercially available plasmids and consists of a consensus 5’splice site (5’SS) motif that is followed, not by other intronic markers (branch point and 3’splice site motif), but rather a 3’processing signal. Previously, it had been shown that such aberrant configurations inhibited proper 3’cleavage and polyadenylation [21–25]. In addition, our data indicates that this element prevents the nuclear export and promotes the degradation of the mRNA. We also observe that mature mRNAs containing a 5’SS build up in nuclear speckles, although it is not clear if this is linked to nuclear retention. By analyzing all human mRNAs, we find that consensus 5’SS are somewhat depleted from 3’UTRs and naturally intronless genes. However, despite this depletion, they are present in almost 6% of all annotated human 3’UTRs from mature mRNAs. Moreover, their level is higher in lncRNAs and other intergenic RNAs. The widespread presence of these elements may indicate that consensus 5’SS motifs may help to down regulate the expression of many genes, and perhaps be used to identify certain ncRNAs, intergenic transcripts and misprocessed mRNAs, and thus prevent their export to the cytoplasm.

## Results

### An element present in the vector backbone of pcDNA3-V5-His disrupts the cytoplasmic accumulation of mRNA

Previously, we demonstrated that *in vitro* transcribed *ftz* mRNAs, are efficiently exported to the cytoplasm if they contain an ALREX-promoting SSCR or an intron [10,26]. Although this result supported the idea that splicing also promotes efficient nuclear export [9], it was unclear why *ftz* mRNAs generated from a plasmid that contained an intron-containing *ftz* gene were not efficiently exported in transfection experiments [10]. It was also unclear why intronless forms of *ftz* in certain studies were well exported (for example [27]). Upon closer inspection, we noticed that the exported mRNAs were transcribed from genes that were inserted into a slightly different plasmid from those versions of *ftz* that were not exported. In particular, any *ftz* gene that was inserted into the pcDNA3-V5-His plasmid (Invitrogen) produced mRNA that was not exported. In contrast, other *ftz* genes which were inserted into the pcDNA3.1+ or pcDNA3.0 plasmids produced mRNAs that were efficiently exported. The pcDNA3-V5-His plasmid contains a region downstream of the multi-cloning site that encodes a V5 epitope tag followed by six histidines and a stop codon.

**Fig 1:**
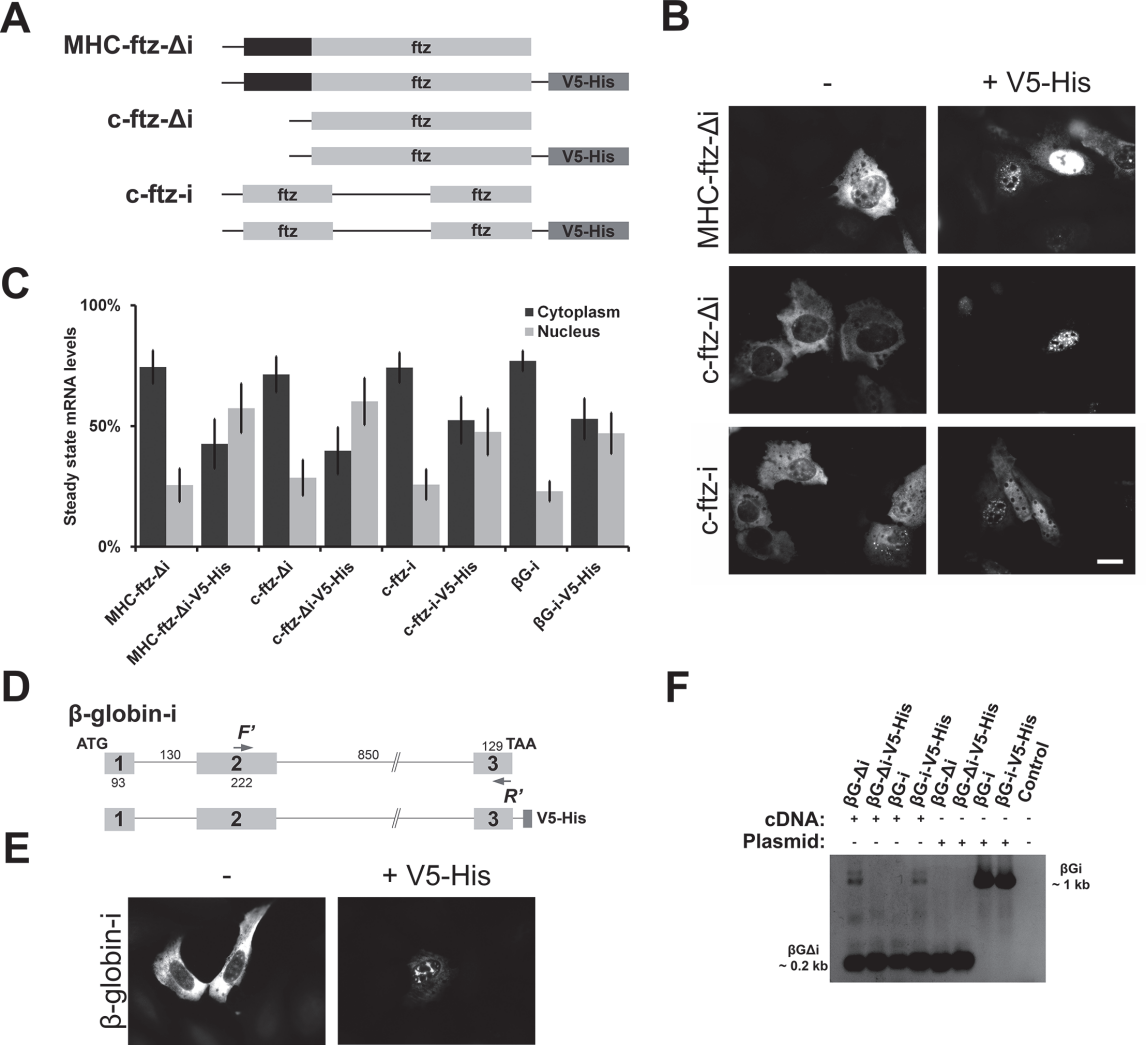
The *V5-His* sequence contains the RNA element that alters the steady state distribution of multiple transcripts. (A) Schematic showing different versions of the *ftz* minigene used in this study. *MHC-ftz-Δi* lacks an intron (Δi) and contains a signal sequence coding region (SSCR) derived from a mouse MHC gene. Versions of this mRNA that lack the MHC SSCR with (*c-ftz-i*) and without an intron (*c-ftz-Δi*) are also shown. When present, the *V5-His* element is present downstream of the stop codon and is thus located in the 3’UTR. (B-C, E) Plasmids containing the various versions of *ftz* (B-C) or *β-globin-i* (C,E) were transfected into U2OS cells and allowed to express for 18 to 24 hours. Cells were then fixed and *ftz* or *β-globin* mRNA was visualized by FISH using specific probes. Example of cells are shown in B and E (Scale bar = 20μm). Quantifications of the steady state distribution of each mRNA in the cytoplasm and nucleus are shown in (C). Each bar consists of the average and standard error of at least three independent experiments, each experiment consisting of the average of >60 cells. (D) Schematic showing the two versions of the *β-globin-i* gene used in this study. Position of forward (“F’”) and reverse (“R’”) primers used in the PCR experiments in F are indicated. As with the *ftz* constructs, the *V5-His* element was present in the 3’UTR (i.e., between the stop codon and the 3’cleavage signal). (F) cDNA was generated from transfected cell lysates and used in a PCR reaction to monitor splicing of the 3’exon (see schematic of *β-globin-i* to for the regions amplified by the primers). Plasmids were used in control PCR reactions. To control for non-specific amplification, DNA was omitted in the last PCR reaction (“control’). PCR reactions were separated by agarose gel electrophoresis and stained with ethidium bromide. Shown here is the grey-scale inverted image. Note that cDNAs generated from cells transfected with *β-globin-i* plasmids were efficiently spliced, even when it contained the *V5-His* element.

To test whether the insertion of genes into pcDNA3-V5-His resulted in the inhibition of export we inserted the *MHC-ftz-Δi* gene, which contains a major histocompatibility (MHC) signal sequence coding region (SSCR) and lacks any intron (Δi), into both plasmids (Fig 1A), with the V5-His encoding sequence being present in the 3’UTR (i.e. after the *ftz* stop codon but upstream of the 3’cleavage signal). Note that the MHC SSCR not only promotes the export of microinjected *in vitro* synthesized mRNA, but also encodes a signal sequence, which targets proteins to the secretory pathway [28]. We also inserted a version of *ftz* that lacks an SSCR and thus encodes a cytoplasmic version of the ftz protein (*c-ftz*) with (i) and without (Δi) an intron into these two plasmids. We then transfected these various constructs into human osteosarcoma (U2OS) tissue culture cells and 18–24 hours later monitored the distribution of mRNA by fluorescent in situ hybridization (FISH). As we had suspected, both *MHC-ftz-Δi* and *c-ftz-Δi* produced from the pcDNA3-V5-His plasmid had mostly a nuclear distribution, while the versions produced from the pcDNA3.1 plasmid were mostly cytoplasmic (Fig 1B and 1C).

To determine whether this phenomenon was specific to the *ftz* gene, we inserted the human *β-globin* gene into both plasmids (Fig 1D), transfected these into cells, and monitored the distribution of the *in vivo* transcribed mRNA by FISH. Since *β-globin* mRNA requires splicing for efficient nuclear export [11,13], we used a version of the gene that contains the two endogenous *β-globin* introns (*βG-i*). As with *ftz*, the distribution of *β-globin-i* expressed from the pcDNA3-V5-His plasmid was more nuclear than the version expressed from pcDNA3.1 (Fig 1E, for quantifications see Fig 1C).

The region encoding the V5-His tag is situated in the 3’UTR of these constructs, upstream of a *bovine growth hormone* (*BGH*) *polyadenylation* sequence. Thus this region is not translated within the context of our *ftz* and *β-globin* reporter mRNAs. We confirmed that the V5-His coding region is incorporated into the mature *c-ftz-Δi* transcript and that the BGH 3’cleavage and polyadenylation site was being used by 3’rapid amplification of cDNA ends (3’RACE) (data not shown). To determine whether the *V5-His* sequence was disrupting splicing of neighbouring introns we isolated cDNA from cells transfected versions of *β-globin* that either contained (i) or lacked (Δi) introns, or the *V5-His* element, and amplified regions flanking the second intron (Fig 1F, see Fig 1D for the region amplified by the forward (F’) and reverse (R’) primers). Although this intron was efficiently amplified from plasmid DNA that contained *β-globin-i* genes, it was not amplified from cDNA isolated from U2OS cells transfected with *β-globin-i* plasmids, regardless of whether the plasmid had the *V5-His* sequence. Thus we concluded that the presence of this sequence did not grossly affect splicing of the nearby intron.

**Fig 2:**
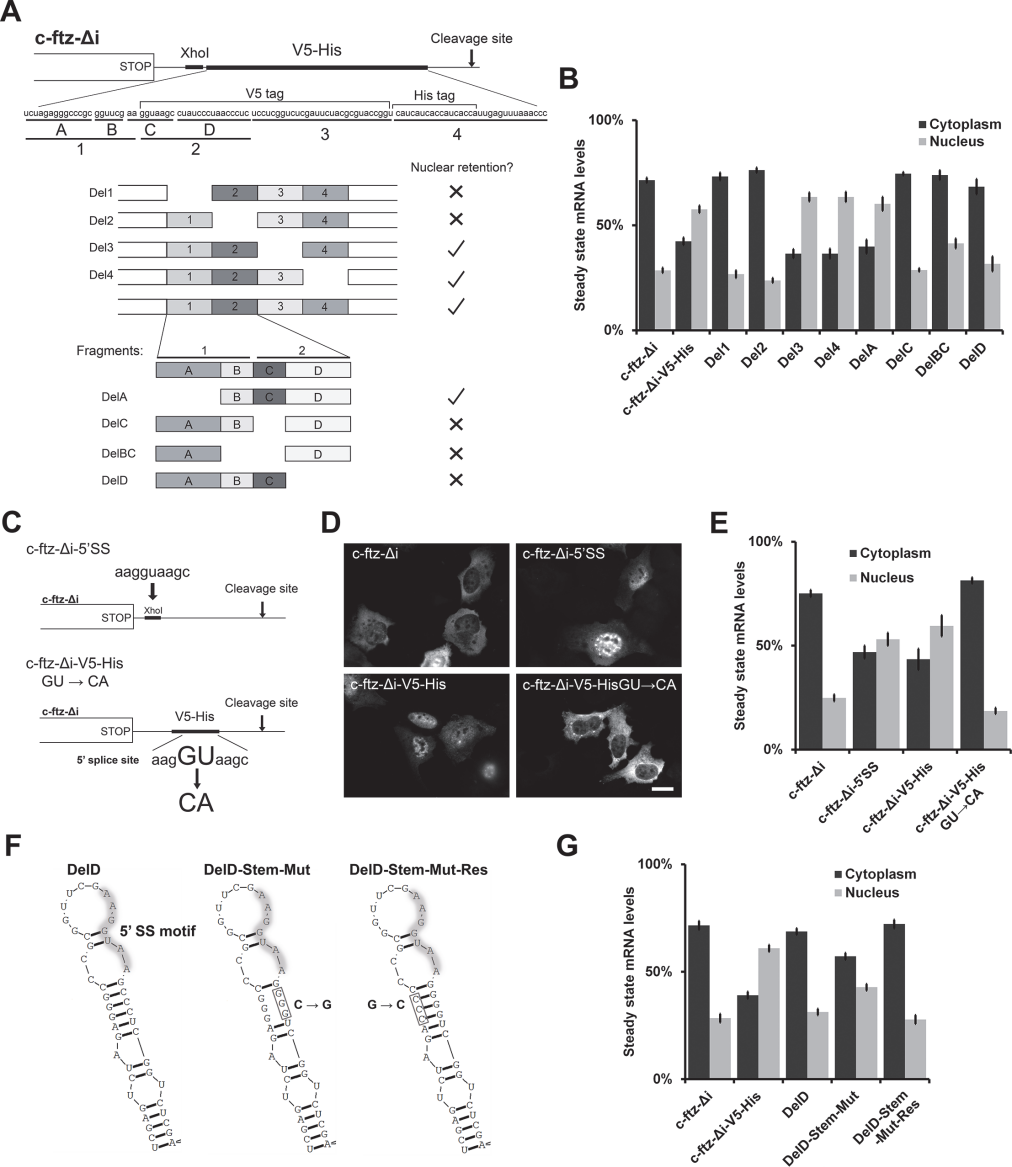
Mapping the minimal sequence in the *V5-His* element that disrupts cytoplasmic mRNA accumulation. (A) Schematic of deletion mutants used to map the element responsible for the nuclear retention activity in the *V5-His* element. (B) U2OS cells were transfected with versions of *c-ftz-Δi-V5-His* gene lacking the various regions indicated in (A), and mRNA distribution was determined as in Fig 1C. Each bar is the average and standard error of at least three independent experiments, each experiment consisting of the average of at least 60 cells. (C) Schematic of constructs designed to test whether the 5’SS motif is responsible for nuclear retention. A consensus 5’SS motif was inserted into the 3’UTR of *c-ftz-Δi* to generate *c-ftz-Δi-5’SS* (top). The central GU nucleotide in the 5’SS motif was mutated to CA in *c-ftz-Δi-V5-His* to generate *c-ftz-Δi-v5-His-GU→CA* (bottom). (D-E) U2OS cells were transfected with the indicated versions of *ftz* and mRNA distribution was determined as in Fig 1C. Examples of *ftz* FISH images are shown in (D) and quantification of their distributions is shown in (E). Scale bar = 20μm. Each bar in E is the average and standard error of at least three independent experiments, each experiment consisting of the average of at least 60 cells. (F) Predicted secondary structure of the region surrounding the 5’SS motif (highlighted) in the *c-ftz-Δi-V5-His-DelD* construct and in the two stem-loop mutants. The RNA structures were generated using RNAStructure 5.03 [57]. (G) U2OS cells were transfected with the indicated versions of *c-ftz-Δi-v5-His DelD* and mRNA distribution was determined as in Fig 1C. Each bar is the average and standard error of at least three independent experiments, each experiment consisting of the average of at least 60 cells.

From these results we conclude that a region in pcDNA3-V5-His interferes with proper mRNA nuclear export and/or stability. We also conclude that although certain SSCRs promote the export of *in vitro* synthesized *ftz* mRNA that was microinjected into cell nuclei, it is not required for the export of *in vivo* transcribed *ftz*. Indeed, this result is quite surprising, as *ftz* has long been used by our lab and others to study mRNA nuclear export, however it is likely that these results were confounded by the presence of this extra sequence. Fortunately, *in vitro* transcribed *ftz* mRNAs do not contain this element as these are generated using plasmids that are linearized using the XhoI restriction site, which is present upstream of the *V5-His* element (see Fig 2A), thus our previous results with mRNA injections still hold.

### The region encoding the V5-His tag contains a 5’splice site motif that disrupts the cytoplasmic accumulation of mRNA

In order to map the region in the *V5-His* sequence that was disrupting proper mRNA distribution, we aligned the two plasmids and discovered that the only differences were present in a stretch of 104 base pairs. This region includes not only sequences that encoded the V5 epitope and His tags, but also parts of a multi-cloning site (Fig 2A). Starting with the *c-ftz-Δi* in pcDNA3-V5-His plasmid, four deletion constructs were made (Del1-4, Fig 2A). These constructs were transfected into cells, and the distribution of the mRNA was monitored by FISH. Quantification of the cytoplasmic and nuclear distributions of the mRNA indicated that the deletion of either of the first two fragments (Del1, Del2) restored the cytoplasmic distribution of *c-ftz-Δi*, but that the deletion of other regions (Del3, Del4) had no effect (Fig 2A and 2B). We then made further deletions within the region that spans deletions 1 and 2 (Del A-D, Fig 2A) and tested the resulting constructs. Deletion of either fragments B, C or D, but not A, restored the cytoplasmic distribution of *ftz* (Fig 2A and 2B).

Upon close inspection of this region we discovered a 5’splice site (5’SS) motif which spanned fragments B and C. 5’SS are recognized by the spliceosome and define the end of an exon and the start of intronic sequence. Normally, a 5’SS must be followed by a 3’SS to delineate the presence of the intron’s boundaries. If however a strong 5’SS is present upstream of a 3’cleavage signal, thus placing the site in the terminal exon, the 5’SS inhibits proper 3’end processing and downregulates gene expression [21–25]. To test whether this motif was responsible for the alterations in mRNA distribution we created two further constructs. First we inserted the 5’SS motif into the 3’UTR of the *c-ftz-Δi* gene that was cloned into pcDNA3.1 to create *c-ftz-Δi-5’SS* (Fig 2C). This insertion alone inhibited the accumulation of *ftz* into the cytoplasm (compare *c-ftz-Δi* to *c-ftz-Δi-5’SS*; Fig 2D, see quantifications in Fig 2E). Second, we disrupted the 5’SS motif in the *c-ftz-Δi-V5-His* construct. This was accomplished by mutating the GU dinucleotide at the center of the motif to CA (*c-ftz-Δi-V5-His-GU→CA*; Fig 2C), a mutation that has been shown to disrupt the recognition of the 5’SS by U1 snRNA [29]. This mutation also restored the cytoplasmic distribution of *ftz* (compare *c-ftz-Δi-V5-His* to *c-ftz-Δi-V5-His-GU→CA*; Fig 2D and 2E).

From these results we conclude that the presence of a 5’SS motif in the 3’UTR inhibits the export and/or promotes the degradation of mRNAs being produced from the pcDNA3-V5-His plasmid.

The only data point that we could not fully explain was the DelD mutant which is exported despite the fact that it contains a 5’SS motif. We suspect that in this particular construct the region surrounding the motif can form a stem loop (Fig 2F) which may prevent the accessibility of this motif. In support of this idea a DelD construct containing three point mutants in the stem (DelD-Stem-Mut; Fig 2F) partially rescued the nuclear retention phenotype (Fig 2G). When the stem was restored with three complimentary mutants (DelD-Stem-Mut-Res; Fig 2F), the resulting mRNA was distributed into the cytoplasm.

These experiments lend support to the idea that 5’SS motif in the 3’UTR must be accessible to in order to affect the distribution and/or stability of the mRNA.

### The 5’SS motif promotes mRNA decay and nuclear retention

**Fig 3:**
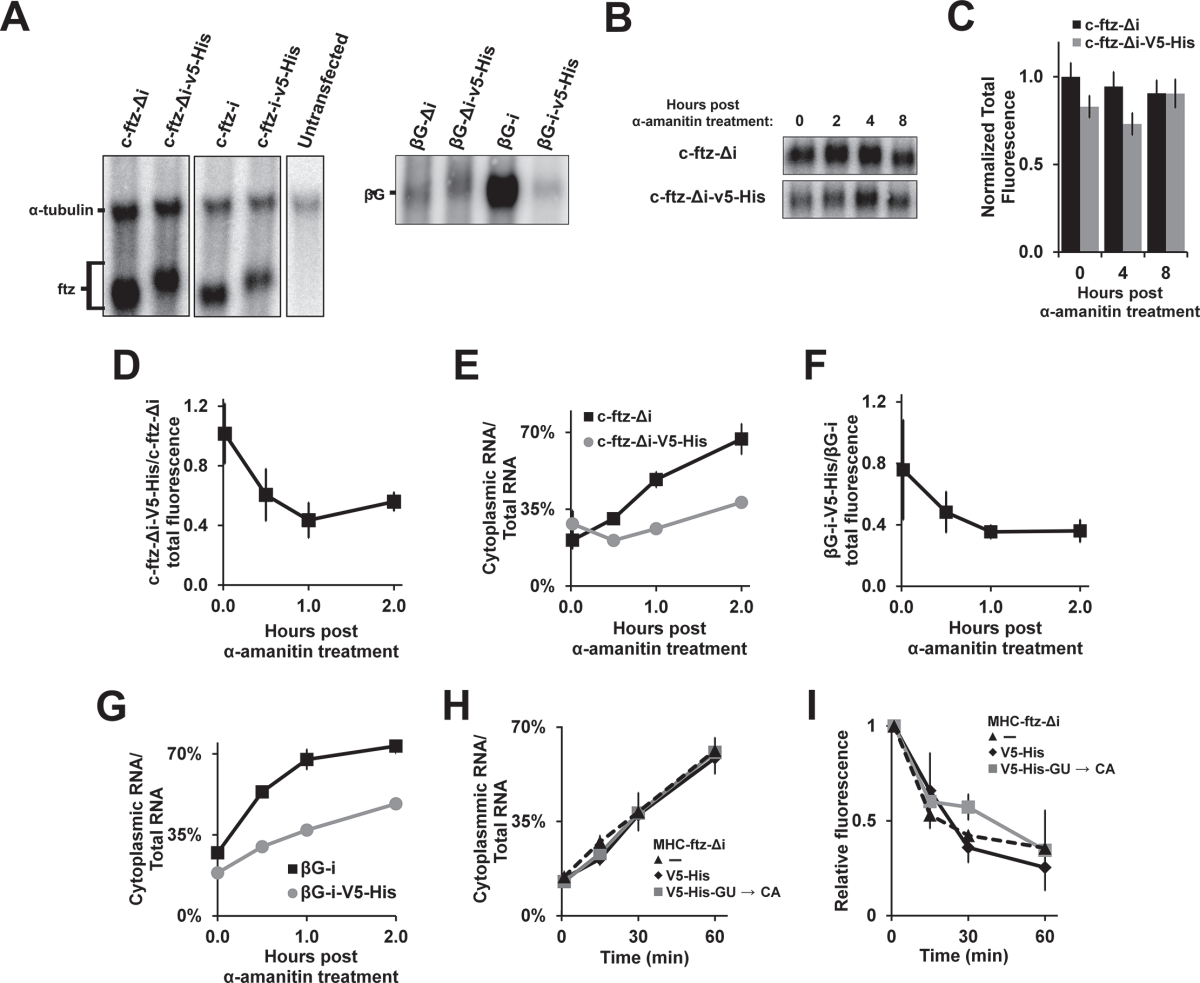
The 5’SS motif decreases mRNA stability and promotes nuclear retention. (A) U2OS cells were transfected with various *ftz* and *β-globin* constructs. 18–24 hrs post transfection, RNA was isolated, separated by agarose gel electrophoresis and probed with radiolabelled probes against α*-tubulin*, *ftz* and *β-globin* mRNAs. Note that the mRNAs containing the *V5-His* element were slightly larger, likely due to the presence of this extra sequence. (B-C) Transfected cells were treated with α-amanitin, at a concentration that completely inhibits transcription (see [30]) for various amounts of time in order to determine the half-life of the mRNA. Levels of *ftz* mRNA were monitored by northern blot (B) or FISH (C). Each bar in C represents the average and standard error of at least 60 cells. (D-E) Plasmids containing *c-ftz-Δi* with and without the *V5-His* element were microinjected into the nuclei of U2OS cells. After allowing mRNA synthesis for 20 min, cells were treated with α-amanitin and mRNA levels were monitored over time by FISH. (D) To determine whether the *V5-His* sequence promotes the degradation of a subset of newly synthesized mRNAs the ratio of *c-ftz-Δi* and *c-ftz-Δi-V5-His* were plotted over time. Each point is the average and standard error of five independent experiments, each of which consist of 15–30 cells. Note that the relative level of *c-ftz-Δi-V5-His* decreases over the first 60 min until 40–50% of the mRNA remains, after which point the ratio is stable. (E) To determine whether the *V5-His* element inhibits nuclear export the percentage of cytoplasmic mRNA was plotted over time. Again, each point is the average and standard error of five independent experiments, each of which consist of 15–30 cells. Note that at the 1 hr time point, at which point most 5’SS-promoted decay has already occurred, the large fraction of nuclear mRNA (representing 70% of the total mRNA) is not efficiently exported. (F-G) Experiments were performed as in (D-E) except the microinjected plasmids contained *βG-i*, with and without the *V5-His* element, and α-amanitin was added 30 min post-injection. The ratio of total *βG-i-V5-His* to *βG-I* FISH signal (F) and the percentage of cytoplasmic mRNA (G) was plotted over time. (H-I) *MHC-ftz-Δi* mRNA, lacking the *V5-His* element (“-”), or containing either the original or mutant version of the *V5-His* element, were synthesized, capped and polyadenylated *in vitro*. The mRNAs were them microinjected into nuclei and the mRNA export (F) and total fluorescence (G) was monitored over time by FISH. Each point is the average and standard error of three independent experiments, each of which consist of 15–30 cells.

Previously it had been reported that the presence of a 5’SS motif in the terminal exon inhibited proper 3’processing and polyadenylation and resulted in the destabilization of the mRNA [21–24]. We first examined the steady state levels of the various *ftz* and *β-globin* reporters +/- the *V5-His* sequence by northern blot (Fig 3A). While the *V5-His*-containing mRNAs were marginally longer than those lacking the element, this was consistent with the presence of an additional 104 nucleotides in their 3’UTRs. Indeed, cDNAs from both *V5-His* containing and lacking mRNAs had the same 3’end as determined by 3’RACE (data not shown). Thus, although the 5’SS may promote 3’cleavage defects, most of the mRNAs analyzed at steady state were cleaved using the same site. It is possible that many RNAs with 3’cleavage defects were degraded. This may explain why the level of spliced *β-globin (βG-i)* was markedly decreased when it contained the *V5-His* sequence (Fig 3A). In contrast, the levels of intronless *β-globin* mRNA *(βG-Δi)*, which is already unstable, was not as affected by this element. Interestingly, the levels of *ftz* mRNA reporters were only slightly decreased by the presence of a *V5-His* sequence. To get a more direct measurement of mRNA half-life, we treated transfected cells with the transcriptional inhibitor α-amanitin at levels which completely block transcription of microinjected plasmids [30], and monitored the amount of *ftz* mRNA remaining after various time points. Contrary to our expectations, we did not see a drastic decrease in the levels of *c-ftz-Δi-V5-His* over time regardless of whether we examined the RNA by northern blot (Fig 3B) or FISH (Fig 3C).

It was still unclear why the overall levels of *c-ftz-Δi-V5-His* was lower than *c-ftz-Δi* (Fig 3A). We hypothesize that perhaps a fraction of the newly synthesized *c-ftz-Δi-V5-His* was unstable, while another fraction avoided this quality control step. Since this second stable fraction would tend to build up over time, it would be expected to be the predominant species present at steady state. To determine the half-life of newly synthesized mRNA, plasmids were microinjected into the nuclei of U2OS cells and the levels of both *c-ftz-Δi-V5-His* and *c-ftz-Δi* were monitored over time after transcriptional shut off. By plotting the ratio of *c-ftz-Δi-V5-His* to *c-ftz-Δi* levels by FISH we estimated how the *V5-His* element influenced mRNA decay. Indeed, we found that the level of *c-ftz-Δi-V5-His* dropped by half after the first hour of α-amanitin-treatment (Fig 3D). After this point the ratio between the two mRNAs leveled off. These observations support the idea that a fraction of newly synthesized *c-ftz-Δi-V5-His* is degraded while a second fraction evades this decay event. When the cytoplasmic to nuclear levels of these transcripts in the previous experiment were plotted over time, the fraction of *c-ftz-Δi* mRNA present in the cytoplasm steadily increased over time (Fig 3E). In contrast, *c-ftz-Δi-V5-His* was not efficiently exported. Moreover, despite the buildup of stable mRNA in the nucleus at the 1 hr time point, the rate of export between 1–2 hrs was similar to that seen with *c-ftz-Δi*, which had much less substrate (i.e. nuclear mRNA) during the same period (1–2 hrs). Since most of the unstable pool was already degraded between 1–2 hrs, our data indicated that the *c-ftz-Δi-V5-His* mRNA that evaded quality control was not efficiently exported. We obtained very similar results when we repeated these experiments with *βG-i* and *βG-i-V5-His* (Fig 3F and 3G).

Next we wanted to test whether the element inhibits export, and/or promotes RNA decay of *in vitro* generated mRNA. Because microinjected *ftz* mRNA requires an ALREX-promoting SSCR (such as the MHC SSCR) to potentiate export, we generated RNA by *in vitro* run off transcription using the *MHC-ftz-Δi-V5-His* plasmid that was linearized with AgeI, which cuts downstream of the *V5-His* element. As controls we generated RNA from the same plasmid, but linearized by XhoI, which cuts upstream of the *V5-His* element. We also generated mRNA from *MHC-ftz-Δi-V5-His-GU→CA* plasmid (linearized with AgeI). These RNAs were then capped and polyadenylated *in vitro* and then microinjected into the nuclei of U2OS cells. Nuclear export of the injected RNA was monitored over time using FISH as we had done previously (see [30]). We found that the 5’SS motif had no effect on the export (Fig 3H) or decay (Fig 3I) of this RNA when compared to either the original *MHC-ftz-Δi* or to the GU to CA mutant.

From these experiments we conclude that the 5’SS motif likely has two distinct activities. First, it promotes the degradation of a subset of the mRNA, and this may be linked to improper 3’end cleavage and polyadenylation. Second, there appears to be a fraction of this mRNA that evades degradation, but is nonetheless retained within the nucleus. Our results with microinjected mRNA would suggest that nuclear retention may be coupled to the transcription and/or processing of the mRNA, despite the fact that most of the retained RNA is properly spliced (Fig 1F) and cleaved (as determined by 3’RACE).

### Characterization of the nuclear retained mRNP-state

**Fig 4:**
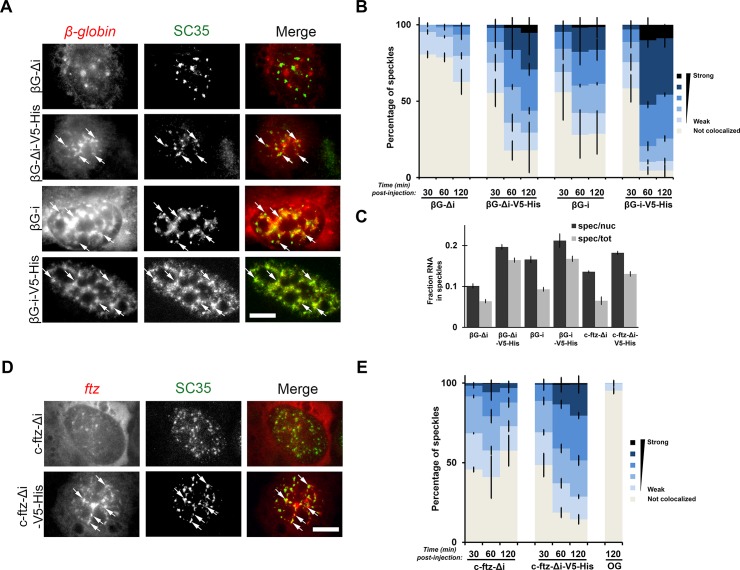
Characterizing the nuclear retained mRNP state. (A) Plasmids containing the indicated genes were microinjected into nuclei. After 2 hrs, cells were fixed and stained for *β-globin* mRNA by FISH and SC35 by immunofluorescence. Each row represents a single field of view. The color overlay shows mRNA in red and SC35 in green. Speckles containing mRNA are indicated by0020arrows (Scale bar = 10μm). (B) Microinjected cells were analyzed for the distribution of mRNAs into nuclear speckles. Individual nuclear speckles, as determined by SC35 staining were analyzed for mRNA content by Pearson Correlation Analysis. For details on the analysis please see the methods section. Each bar is the average and standard error of the mean of three experiments, each of which consist of 150–200 speckles analyzed from 15–20 cells. Note that nuclear-speckle association is enhanced by the *V5-His* element even in the presence of splicing. (C) The amount of various mRNA present in nuclear speckles (as defined by the brightest 10% pixels in the nucleus, using SC35 immunofluorescence—see methods section and Akef et al., 2013 for details) as a percentage of either the total nuclear (“spec/nuc”) or total cellular (“spec/tot”) mRNA level in cells 1 hr post-microinjection. Each data point represents the average and standard error of the mean of 10–20 cells. Note that *β-globin-Δi* was not enriched in speckles as ~10% of the nuclear RNA fluorescence was present in these regions, which represents 10% of the total nuclear area. (D-E) Similar to (A-B), except that the co-localisation of *c-ftz-Δi* +/- *V5-His* RNA with nuclear speckles was analysed. As a control, the colocalization of SC35-positive speckles with microinjected 70kD dextran conjugated to oregon green dye (“OG”) was analyzed.

We noticed that nuclear retained *c-ftz-Δi-V5-His* and *β-globin-i-V5-His* mRNAs formed distinct foci in the nucleus and we wondered if these colocalized with nuclear speckles. Nuclear speckles are foci that contain RNA polymerase II, splicesomal components, various splicing factors (such as SR proteins) and mRNA export factors [31]. Certain mRNAs can be targeted to speckles, either by post-transcriptional splicing events [14,32] or by yet-to-be identified motifs present in many mRNAs [13]. Since splicing promotes the efficient nuclear-speckle targeting of *β-globin* [13], we decided to investigate the localization of newly synthesized *β-globin-Δi* with or without the *V5-His* element. We microinjected plasmids containing the various genes into the nuclei of U2OS cells and the newly synthesized RNA was visualised by FISH staining at various time points post-injection. Nuclear speckles were imaged using immunofluorescence directed towards the protein SC35, a speckle marker [33,34]. We then performed Pearson Correlation Analysis, as previously described (see [13]), and found that the *V5-His* fragment promoted nuclear speckle-targeting of *β-globin-Δi* (Fig 4A and 4B). When the *V5-His* sequence was incorporated into *β-globin-i* it enhanced nuclear speckle-targeting above what splicing alone promoted (Fig 4A and 4B). Association of the various mRNAs with nuclear speckles happened within the first hour of expression (Fig 4B) and persisted over the next hour. The amount of total mRNA in the speckles also increased in the presence of the *V5-His* element, regardless of whether the mRNA was spliced (*i*) or not *(Δi*) (Fig 4C). The targeting of *β-globin-Δi* to nuclear speckles was also seen in transfected cells (S1 Fig). We then repeated these experiments with *c-ftz-Δi* and *c-ftz-Δi-V5-His*. As reported previously (see [13]), *c-ftz-Δi* has a natural propensity to associate with nuclear speckles, but this increased with the presence of the *V5-His* element (Fig 4C–4E).

From these experiments we concluded that the 5’SS motif promotes the accumulation of mRNAs into nuclear speckles. Whether or not that these mRNAs are actively retained and/or degraded at these sites remains to be determined.

### The 5’SS motif does not prevent the loading of UAP56 or TAP onto mRNA

**Fig 5:**
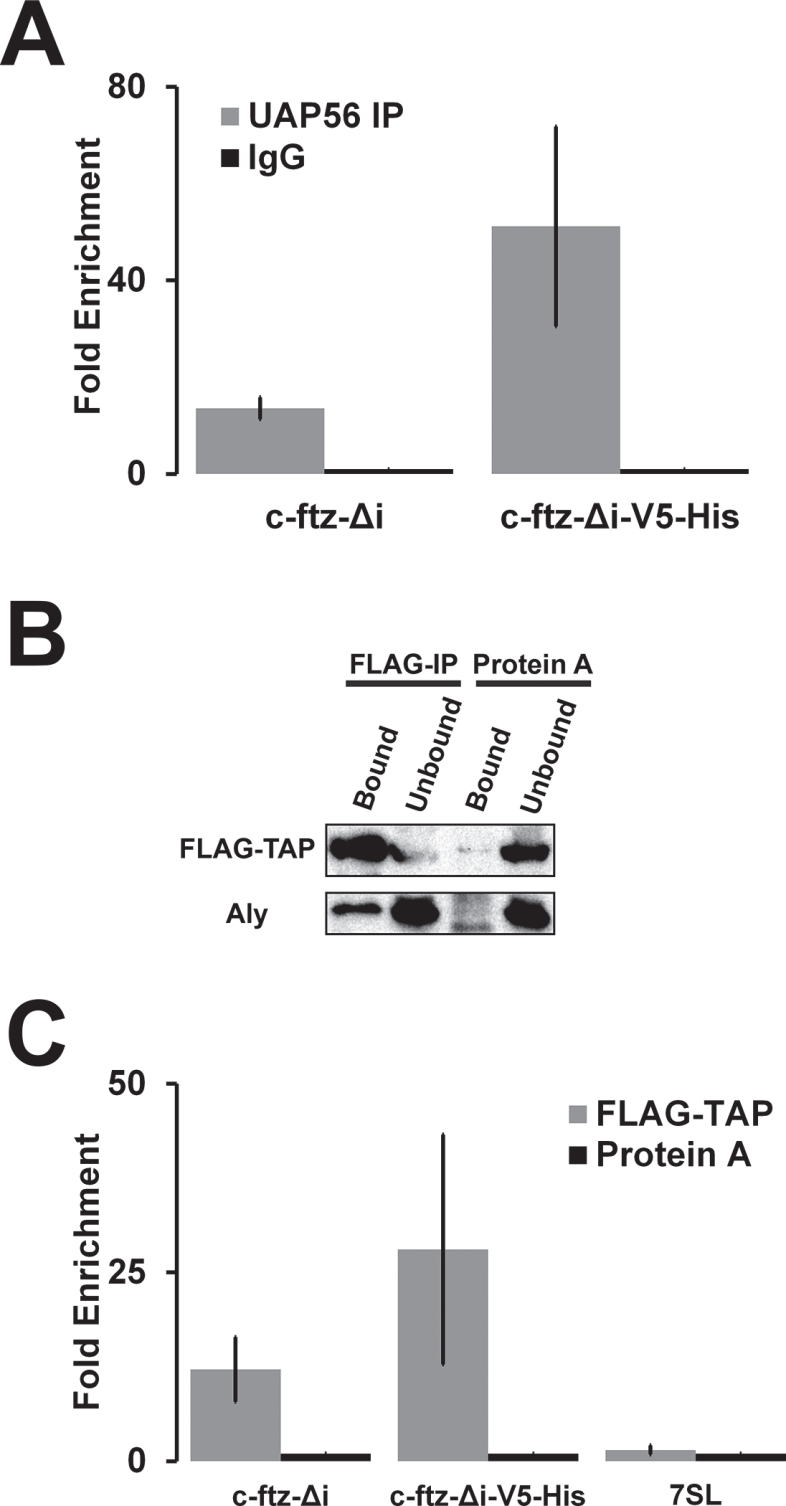
The *V5-His* element does not prevent the association of UAP56 with mRNA. (A) U2OS cells were transfected with various *ftz* constructs. 18–24 hrs post transfection cell lysates were collected and UAP56 was immunoprecipitated with a rat monoclonal anti-UAP56 antibody or pre-immune rat IgG. RNA levels were measured by quantitative RT-PCR as previously described [13]. Each bar is the average and standard error of three independent experiments. Note that under the same conditions, we previously showed that other RNAs, such as the ncRNA 7SL, are not enriched in UAP56 RIPs [13]. (B-C) U2OS cells were transfected with plasmids containing the FLAG-TAP fusion gene alone (B) or with various *ftz* constructs (C). 18–24 hrs post-transfection, lysates were collected and protein was precipitated using FLAG M2 beads or control Protein A beads. (B) Eluates from the beads (“Bound”) and 15% of the unbound fraction were separated by SDS-PAGE and probed by western blot for FLAG and for Aly, a known TAP-associated protein. (C) RNA from the precipitates was isolated and converted to cDNA using random hexanucleotides and the levels for either *ftz* constructs or the 7SL ncRNA were measured by quantitative RT-PCR as previously described [13]. Each bar is the average and standard error of four independent experiments.

Previously we determined that the nuclear export of *ftz* is dependent on UAP56 and URH49 [13], two RNA helicases that are part of the TREX complex [35]. Furthermore we demonstrated that UAP56 associates with *MHC-ftz*, to the same degree, regardless of whether or not it contained an intron [13]. Although we had presumed that this was due to the action of ALREX-promoting SSCRs, it had previously been noted that intronless *ftz* lacking SSCRs could recruit UAP56 and Aly [36]. Since UAP56/URH49 depletion prevented the egress of mRNAs from nuclear speckles [13], we considered whether mRNAs containing the *V5-His* element failed to recruit UAP56, thus trapping the transcript in speckles. To determine whether the 5’SS motif inhibited export by preventing UAP56 association we performed RNA-immunoprecipitation (RIP) experiments with anti-UAP56 antibodies, and detected the presence of mRNA by performing quantitative real time PCR, as we had done previously [13]. We found that both *c-ftz-Δi-V5-His* and *c-ftz-Δi* were present at high levels in the UAP56 immunoprecipitates (Fig 5A). Interestingly, the *V5-His* element appeared to enhance UAP56-association. We also obtained the same result when we repeated these experiments in cells treated with formaldehyde prior to lysis (data not shown), which cross-links RNAs and proteins, thus preventing the formation of RNA-protein complexes in the post-lysis lysate [37]. Using the same procedure, we previously demonstrated that a highly abundant control non-coding RNA, 7SL, does not co-precipitate with UAP56 [13].

A number of studies have indicated that UAP56 must be displaced from the mRNA in order to promote the recruitment of TAP (also known as Nxf1), a nuclear transport receptor, with the mRNA [38]. In light of this we monitored whether TAP associated with *c-ftz-Δi-V5-His* and *c-ftz-Δi*. As we lack antibodies to TAP, we performed RIP assays using exogenously expressed FLAG-TAP. This assay has been used by other groups to monitor TAP association with various RNAs [16,39,40]. As Aly is found within the FLAG-TAP immunoprecipitate (Fig 5B), we were confident that mRNPs were likely conserved during the protocol. To our surprise we found that both *c-ftz-Δi-V5-His* and *c-ftz-Δi* were present at high levels in the FLAG-TAP immunoprecipitates (Fig 5C). Again, the *V5-His* element appeared to enhance FLAG-TAP association. In contrast, 7SL, which uses a CRM1-dependent export pathway [41], was not enriched in the FLAG-TAP immunoprecipitates (Fig 5C).

From these experiments we conclude that the 5’SS motif does not prevent nuclear export by disrupting the association of either UAP56 or TAP with the mRNA. Instead, it is likely that this motif recruits additional factors to the mRNP which actively retains the mRNA in the nucleus and thus preventing TREX complex members and TAP from engaging the nuclear pore.

### The 5’SS motif is depleted at the beginning and end of 3’UTRs and 3’exons of protein-coding genes in the human genome

**Fig 6:**
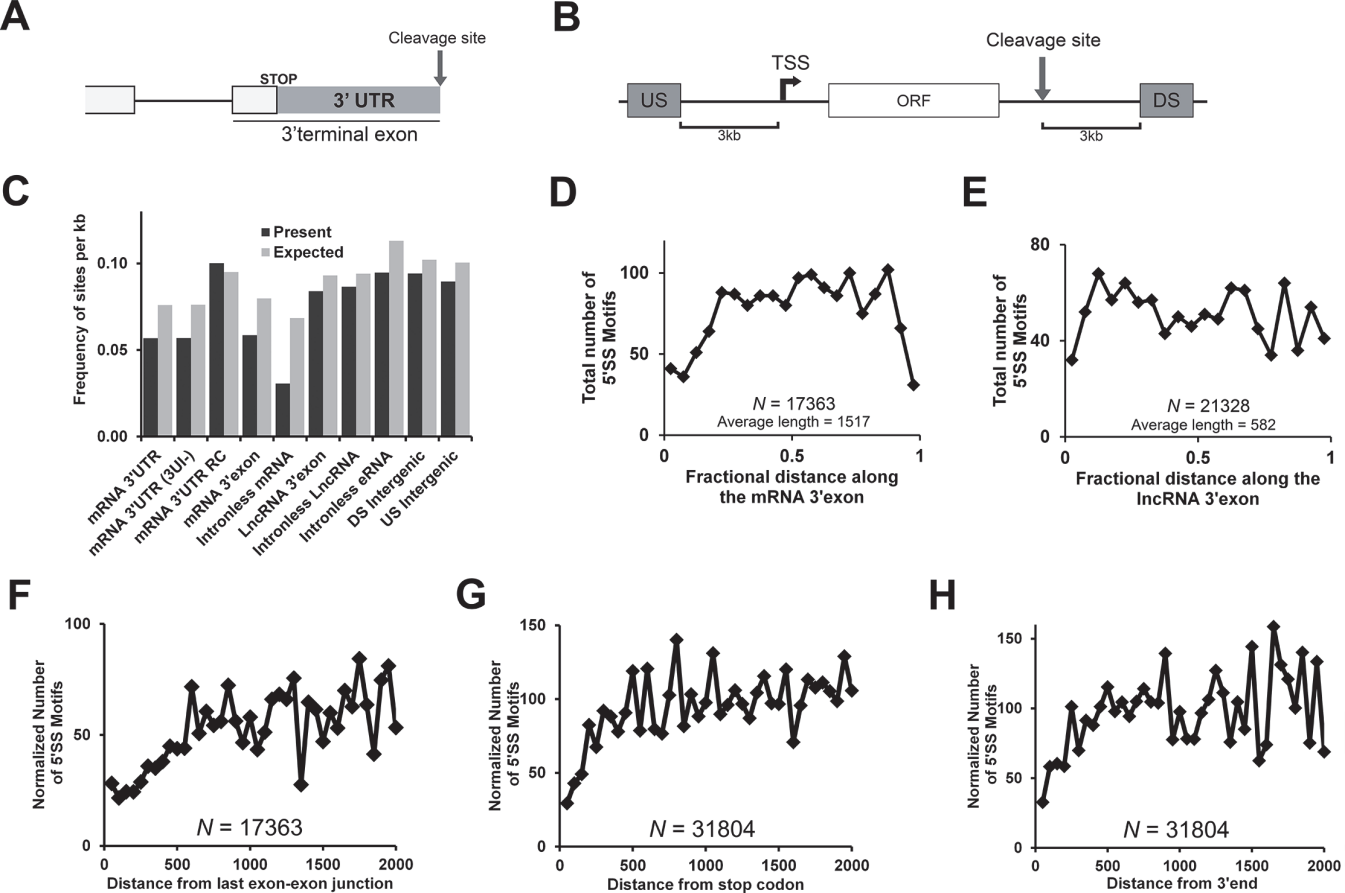
The 5’SS motif is depleted in the 3’exons and 3’UTRs of genes across the human genome. Various regions across the human genome were analyzed for the presence of the consensus 5’SS motif. As controls, we analyzed regions 1kb regions that were 3kb upstream (“US”) from the transcriptional start sites of and downstream (“DS”) from the 3’cleavage site of protein-coding genes as seen in the schematic in (A). Note that introns are known to be rarely present in the 3’UTRs and as a result the 3’UTR is normally present in its entirety in the 3’terminal exon (B). (C) The frequency of the consensus 5’SS motif was analyzed in various loci (see Table 1 for a full description). The expected frequencies were determined using the tri-nucleotide frequencies in each region. Regions analyzed included 3’UTRs from all protein coding genes, 3’UTRs that do not contain introns (“3’UTR (3UI-)”), the reverse compliment of 3’UTRs (“3’UTR RC”), the 3’terminal exon of mRNAs, intronless mRNAs, the 3’terminal exon of lncRNAs, intronless lncRNAs, intronless eRNAs, and upstream and downstream intergenic regions as defined in (A). List of lncRNAs was taken from LNCipedia. List of eRNAs was taken from the FANTOM5 consortia. (D-E) Distribution of the consensus 5’SS motif along the 3’terminal exon for mRNAs (D) and lncRNAs (E). The lengths of each RNA were divided into 20 bins and the number of 5’SS motifs in each bin was plotted. (F-H) The number of 5’SS motifs was plotted as a function of distance from the last exon-exon junction (F), stop codon (G), or 3’cleavage site (H) of mRNAs. The number of 5’SS motifs in each bin of 50 nucleotides was normalized for the total number of mRNAs remaining as distance increases.

We rationalized that if the 5’SS motif, inhibits proper 3’end processing, promotes decay and causes nuclear retention, that it should be depleted in most naturally intronless protein-coding genes from the human genome. Moreover, these motifs should also be eliminated from the 3’exons within human mRNAs. We also reasoned that since 3’UTRs are usually present in their entirety in the 3’exon (see schematic, Fig 6A), they should also be depleted of the 5’SS motifs. Indeed we found that the consensus motif ([C/A]AGGU[C/A]AG) was present in both 3’exons and 3’UTRs at about half the rate at which it appeared in either intergenic regions (upstream, “US”, or downstream, “DS” of protein coding mRNAs, see schematic Fig 6B) or in the reverse compliment of 3’UTRs (“3’UTR RC”; Fig 6C, Table 1). About 5% of all protein-coding genes have introns in their 3’UTRs, however even if these are eliminated (see Fig 6A, “3’UTR 3UI-”; list of human genes with 3’UTR introns was obtained from the Blencowe lab), the remaining 3’UTRs are still depleted of the consensus 5’SS motif (Fig 6C, Table 1). When naturally intronless genes were analyzed (list obtained from the intronless gene database [42], http://www.bioinfo-cbs.org/igd/) these were even more depleted of 5’SS motifs (Fig 6C, Table 1). Since 3’UTRs are AU-rich, they may contain fewer 5’SS motifs due to their nucleotide composition. To control for this effect we compared the frequency that the motif is present to the expected rate that they would appear given the trinucleotide frequencies of these genomic regions. Again 3’exons and 3’UTRs are depleted of the 5’SS consensus motif, but to our surprise the rate of depletion, in comparison to the expected frequency (~30%), was not as dramatic as we thought it would be. The reduction was much more pronounced for intronless genes (>50% depletion).

**Table 1:** The presence of consensus 5’SS motifs in various human loci.

RNA Species/Region	Number Analyzed	Average Length	Median Length	5'SS Motif Depletion
mRNA 3'UTR	31804	1103	596	25.3%
mRNA 3'UTR RC	31804	1103	596	-5.4%
mRNA 3'UTR (3UI-)	29901	1099	592	25.4%
mRNA 3'exon	17363	1517	1001	26.7%
Intronless mRNA	687	1812	1294	55.3%
LncRNA 3'exon	21328	582	301	9.8%
Intronless LncRNA	159	2335	929	8.1%
FANTOM5 Intronless eRNA	5200	464	322	16.3%
Downstream Intergenic	20795	999	999	7.8%
Upstream Intergenic	20795	999	999	11.0%

5’SS Motif depletion is based on the percent decrease between the observed number of motifs and the expected number based on tri-nucleotide frequencies (see Fig 6C).

We next examined other classes of RNAs such as long noncoding RNAs (lncRNAs—list of human lncRNAs obtained from the LNCipedia database, http://www.LNCipedia.org, [43]). Since the act of splicing would naturally eliminate many 5’SS motifs, we analyzed the 3’exons of spliced lncRNAs and intronless lncRNAs. Both of these only displayed a weak depletion of these motifs, on par to what was seen in intergenic regions (Fig 6C, Table 1). This may indicate that either the presence of 5’SS motifs do not impede the activity of lncRNAs, and/or that the list of lncRNAs that we used contains many non-functional transcripts (for a discussion on this topic, see [5]). We also examined enhancer RNAs (eRNAs) which are transcribed from certain enhancer regions [44]. Some of these are thought to bind to the Mediator complex to modulate the bending of DNA, and the formation of topologically associated DNA domains, which serve to promote local transcriptional activation [45]. As described elsewhere, most eRNAs are short and intronless [44]. Again, since splicing would naturally eliminate 5’SS motifs, we restricted ourselves to naturally intronless eRNAs as annotated by the FANTOM5 project [44]. We saw a slight depletion of 5’SS consensus motifs in eRNAs (Fig 6C, Table 1) beyond what we saw for intergenic regions and lncRNAs. This may not only reflect that these motifs may impede eRNA function, but that introns, and thus splicing signals, are eliminated from these regions.

The fact that 5’SS motifs were not completely eliminated from 3’exons of protein-coding genes suggested that perhaps these were only depleted in specific parts of the exon. With this idea in mind, we determined the frequency that this motif appears along the length of 3’exons. As one can see from Fig 6D, 5’SS motifs were depleted in two regions: at the start and end of the exon. When this analysis was repeated for the 3’exon of lncRNAs, this trend was not as obvious (Fig 6E). We then plotted the distance between each 5’SS motif and various land marks in protein-coding genes. We found that the motif was depleted within the first 500 nucleotides after the last exon-exon junction (i.e., the start of the 3’exon) (Fig 6F), within the first 200 nucleotides after the stop codon (Fig 6G) and about 200 nucleotides upstream of the 3’cleavage site (Fig 6H).

From this analysis we conclude that although the consensus 5’SS motif is present at a reduced frequency in 3’exons, it appears to be more heavily depleted downstream from the last exon-exon junction and upstream from the 3’cleavage site. This regional depletion may indicate that these motifs may only act to promote decay and/or nuclear retention when they are at either end of the last exon.

## Discussion

One emerging theme in RNA biology is the requirement for various nuclear quality control mechanisms to prevent the nuclear export of aberrantly processed and intergenic RNA molecules. Here we identify the consensus 5’SS motif as the RNA element that inhibits mRNA nuclear export when present in the terminal exon. As documented by others, this motif also inhibits proper 3’processing and promotes the degradation of mRNAs.

It is likely that these mRNAs are targeted to speckles and retained in the nucleus by the association of U1 snRNP. This complex has been shown to inhibit the expression of other mRNAs containing 5’SS motifs in their terminal exons [21–24]. It is also likely that U1 snRNP-association targets certain pre-mRNAs to speckles in order to complete the splicing reaction post-transcriptionally [32]. It is however unclear how U1 snRNP-association inhibits export of mRNAs that have acquired export factors such as UAP56 and TAP. Tethering of certain splicesome-associated proteins, such as U2AF65 and U1-70K, to an mRNA inhibits protein production [25] and can cause nuclear retention of the mRNA [46]. In yeast, proteins of the nuclear basket are required to block the export of mRNAs with retained introns [47,48] and this may also require additional spliceosome-associated factors [49]. It is thus possible that the metazoan basket proteins can also recognize snRNPs that are still loaded onto the transcript and inhibit RNA nuclear export. Alternatively it is possible that the act of retention within speckles may prevent any engagement of these mRNAs with the nuclear pore.

Other data have implicated the U1 snRNP component U1-70K in the downregulation of genes harboring 5’SS motifs in their terminal exon by inhibiting 3’end cleavage [22]. This explains why the downregulation of U1 snRNA promotes the appearance of premature 3’end processing events in intronic sequences [50]. Finally a recent report has indicated that in yeast, certain 5’SS motifs can initiate a splicing reaction that does not include a re-ligation step. The cleaved mRNA can then be degraded in what has been termed splicing mediated decay (SMD) [51]. Thus it is clear that 5’SS that do not participate in normal splicing reactions, but are instead retained within the transcript, promote a variety of reactions that inhibit mRNA expression.

Our bioinformatics data suggests that 5’SS motifs may only function to inhibit gene expression when they are present near other splice junctions or the 3’cleavage signals indicating that these motifs only function when they are near other landmarks. Thus it is likely that the recognition of these 5’SS may be aided by nearby fully assembled spliceosomes and the 3’processing machinery. Positive interactions between upstream splicing events and downstream 5’SS motif recognition have been documented and these interactions are known to promote the coupling of multiple splicing reaction along an mRNA and help to define proper exon boundaries (i.e., exon definition) [52]. Our results indicate that the lack of accessible 5’SS motifs near the beginning or end of the terminal exon may help to enforce proper terminal exon definition, and by extension proper mRNA definition. Our data suggests that these signals can be overcome not only by the elimination of the motif, but also by the formation of structures that impede the recognition of these motifs by U1 snRNP (see DelD construct Fig 2A–2B and 2F–2G). Indeed this idea is in line with previous work demonstrating that U1-mediated events, be they splicing or mRNA-downregulation, are very sensitive to local secondary structure around the 5’SS motif [25,53,54].

We are currently testing whether components of the spliceosome, 3’end processing machinery and parts of the nuclear pore are involved in the nuclear retention of RNAs harboring 5’SS in their terminal exon. This work will help provide insight into how mature mRNAs, which are depleted 5’SS motifs, are differentiated from other classes of RNAs.

## Material and Methods

### Plasmids constructs and primers

The *MHC-ftz-Δi*, *c-ftz-Δi*, *c-ftz-i*, *βG-Δi* and *βGi* constructs in pcDNA3.0 were described previously [10,13]. To generate the *V5-His* containing plasmids, the reporter gene of interest was digested with HindIII and XhoI and ligated into the pcDNA3.1+ plasmid (containing the V5-His epitope tag-encoding sequence) that was digested with the same enzymes. To generate the deletion mutants, primers flanking the region to be deleted in *c-ftz-Δi-V5-His* were designed and Phusion PCR reactions performed according to the manufacturer’s instructions (Thermoscientific) but using 10–20 ng DNA template. Forward and reverse primers were as follows: Del1 F’ 5’- GGT AAG CCT ATC CCT AAC CCT CT—3’, R’ 5’- CTC GAG TTT AAT AGA AAT TGG GAC AGC AAG—3’, Del2 F’ 5’- CCT CGG TCT CGA TTC TAC GCG TAC CGG—3’, R’ 5’- TTC GAA CCG CGG GCC CTC TAG A—3’, Del3 F’ 5’- CAT CAT CAC CAT CAC CAT TGA GTT TAA AC—3’, R’ 5’- AGA GGG TTA GGG ATA GGC TTA CC—3’, Del4 F’ 5’- GCT GAT CAG CCT CGA CTG TGC C—3’, R’ 5’- ACC GGT ACG CGT AGA ATC GAG ACC—3’, DelA F’ 5’- GGT TCG AAG GTA AGC CTA TCC C -3’, R’ 5’- CTC GAG TTT AAT AGA AAT TGG GAC AGC AAG—3’, DelBC F’ 5’—CTA TCC CTA ACC CTC TCC TCG GTC—3’, R’ 5’- GCG GGC CCT CTA GAC TCG AG—3’, DelC F’ 5’- CTA TCC CTA ACC CTC TCC TCG GTC—3’, R’ 5’- CGA ACC GCG GGC CCT CTA—3’, DelD F’ 5’- CCT CGG TCT CGA TTC TAC GCG—3’, R’ 5’- GCT TAC CTT CGA ACC GCG GG—3’, DelD-Stem-Mut-F’ 5’- CGC GGT TCG AAG GTA AGG GGT CGG TCT CGA TTC TAC GC—3’, DelD-Stem-Mut-R’ 5’- CGC GTA GAA TCG AGA CCG A- 3’, DelD-Stem-Mut-Res-F’ 5’- GTC CCA ATT TCT ATT AAA CTC GAG TCT AGA CCC CCC GCG GTT CGA AGG TAA- 3’, DelD-Stem-Mut-Res-R’ 5’- TTA CCT TCG AAC CGC GGG GGG TCT AGA CTC GAG TTT AAT AGA AAT TGG GAC-3’. The PCR product was treated with DpnI (NEB) at 37 °C for 12–18 hrs and purified using the PCR purification kits (Geneaid). To facilitate DNA ligation, the PCR products was treated with polynucleotide kinase (NEB) for 1 hour at 37 °C, and T4 ligase was subsequently added to the reaction mixture and incubated for 16–18 hrs at 16 °C. PCR reaction using Taq polymerase (NEB) was used to screen for positive ‘hits’ prior to DNA sequencing. The *c-ftz-Δi-5’SS* and *c-ftz-Δi-V5-His-GU→CA* constructs were generated using a site directed mutagenesis PCR reaction using Phusion polymerase and specific primers (for c-ftz-Δi-5’SS, F’ 5’- TCT TGC TGT CCC AAT TTC TAT TAA ATC TAA GGT AAG CAG ACA TGC ATC TAG AGG GCC CTA TTC T -3’, and R’ 5’- TAG GTG ACA CTA TAG AAT AGG GCC—3’; for *c-ftz-Δi-V5-His-GU→CA* F’ 5’- GAG GGC CCG CGG TTC GAA GCA AAG CCT ATC CCT AAC CCT CTC CTC GG -3’, and R’ 5’- CCG AGG AGA GGG TTA GGG ATA GGC TTT GCT TCG AAC CGC GGG CCC TC -3’).

### Cell culture and DNA transfection experiments

Human osteosarcoma (U2OS) and embryonic kidney 293T (HEK293T) cells lines were maintained in DMEM (Wisent) supplemented with 10% fetal bovine serum (Wisent) and penicillin/streptomycin antibiotics (Sigma). Cells were plated overnight on glass coverslips (VWR) in 6 well plates. For all DNA transfections, U2OS cells were transfected with the appropriate amount of DNA plasmid according to the manufacturer’s protocol using LipoD U2OS DNA *in vitro* transfection reagent (SignaGen Laboratories) for 18–24 hrs.

### Microinjection, FISH staining and immunostaining

Microinjection experiments were performed as previously described [10,13,30]. For all microinjection experiments, DNA plasmids or RNA was microinjected at 0.4–1 μg/μl concentration with 70kDa Dextran conjugated to Oregon Green (Invitrogen) and 1X injection buffer (100 mM KCl, 10 mM HEPES pH 7.4). For the mRNA decay experiment, cells were treated with 1 mg/ml α-amanitin (Sigma) for 20 min (*ftz* injections) or 30 min *(β-globin* injections) post-injection to inhibit transcription. Note that the *β-globin-i* construct used in the microinjection experiment had a point mutation near the end of the ORF (A1424C), which is unlikely to have affected the results.

For FISH staining, all cells were washed with PBS, fixed in 4% paraformaldehyde (Electron Microscopy Sciences) in PBS and permeabilized with 0.1% Triton X-100 in PBS (ThermoScientific). Cells were subsequently washed with 60% formamide in 1X SSC buffer (150 mM NaCl, 15mM NaCitrate, pH 7.1) and then incubated overnight at 37 °C in hybridization buffer (60% formamide, 100 mg/ml dextran sulfate, yeast tRNA, 5 mM VRC, 1X SSC) containing 200 nM Alexa 546-conjugated ssDNA probe (Integrated DNA technologies). *Ftz* and *β-globin* probe sequences as previously described [10,13,30]. Subsequently, cells were washed with at least three times with 60% formamide in 1X SSC buffer and the coverslips mounted with DAPI fluoromount G-stain (Southern Biotech). Immunostaining was performed on fixed cells first using the primary antibody mouse monoclonal anti-SC35 (Clone SC35, Sigma; 1:1500–1:2000 dilution), diluted in immunostain solution (PBS, 0.1 mg/ml RNase free BSA). The coverslips were incubated for 30 minutes, washed three times with PBS and incubated with secondary antibody conjugated to Alexa 647 (Molecular Probes) for 30 minutes at 1:2000 dilution. Cells were imaged and the nuclear and cytoplasmic ratios of FISH fluorescence were quantified as described previously [30].

### In vitro RNA synthesis and purification

The constructs were linearized by AgeI (*MHC-ftz-Δi-V5-His* and *MHC-ftz-Δi-V5-His-GU→AC*) or XhoI (*MHC-ftz-Δi*) digestion and precipitated overnight in -20°C with 40 mM KOAc and 2.5X 100% ethanol. *In vitro* transcription and capping reactions were performed using the T7 mMESSAGE mMACHINE transcription kit (Ambion) following the manufacturer’s protocol. The resulting RNA was polyadenylated using the Poly(A) tailing kit (Ambion) to generate poly(A) tails of 200 to 300 nucleotides in length. The *in vitro* synthesized RNA was then purified with Purelink RNA purification kit (Ambion), eluted with 20 μl RNase free water and resuspended accordingly in 10X injection buffer.

#### 3’RACE, RT-PCR, Northern blotting and α-amanitin chase experiments

For 3’RACE and RT-PCR experiments, total RNA was extracted from transfected U2OS cells using either Trizol (Life Sciences) or Purelink RNA purification kit (Ambion) according to the manufacturer’s instructions. ~ 1μg total RNA was used for first strand synthesized using murine MLV reverse transcriptase (Invitrogen) and oligo(dT) primer according to the manufacturer’s instructions. For 3’RACE, experiments, the resulting cDNA was amplified using c-ftz F’ primer 5’- ATG GGG TGT TGT CCC GGC TGT TGT—3’and oligo(dT). The resulting PCR product was cloned into CloneJet (Fermentas) vector following the sticky-end cloning protocol and transformed into DH5α competent cells. DNA was extracted from colonies using Midiprep kit (Geneaid) and sent for sequencing.

To determine if the *V5-His* element affected splicing, primers flanking the second intron of *β-globin-i* were used to amplify cDNA from cell lysates. The sequences of these primers are as follows: F’ 5’—TCG GTG CCT TTA GTG ATC GC—3’, R’ 5’—TTA GTG ATA CTT GTG GGC CAG GG—3’ (see Fig 1D).

For Northern blotting, total RNA was isolated from transfected U2OS cells as for the RT-PCR experiment. The RNA was separated on a 1–1.5% agarose gel in 1X MOPS buffer (22 mM MOPS, 5 mM NaOAc, 0.5M EDTA) with 3% formaldehyde. Capillary action was used to transfer the RNA onto a nitrocellulose member using 20X SSC solution. The blots were pre-hybridized with Church buffer (0.5 M phosphate buffer, pH 7.2, 7% SDS, 1% BSA and 1 mM EDTA) for 4 hrs. Radiolabelled probes oligonucleotides specific for *ftz*, *β-globin* and *tubulin* were generated from DNA templates (reporters in pcDNA3.0 constructs digested with HindIII and XhoI) or PCR products (for tubulin) using [α^32^P]dATP (Perkin Elmer) and the Prime-a-gene labeling system (Promega). The probes were purified using a 1 ml syringe column lined with glass wool and Sephadex-G beads, denatured at 95 °C for 5 minutes, added to the membrane and probed overnight at 65 °C. The membrane was washed for at least four times in wash buffer (2X SSC, 0.1% SDS), exposed on a phosphoimager cassette for 4–72 hrs and imaged with the Typhoon phosphoimager system.

For all α-amanitin chase experiments, 1 mg/ml α-amanitin (Sigma) was added after 18–24 hrs following transfection and total RNA was isolated at various time points post drug treatment as indicated in the Fig 3B and 3C.

#### RNA-IP experiments

The UAP56 RNA-IP experiments were performed exactly as previously described [13], using a rat-polyclonal antibody against UAP56 [55], or rat-preimmune serum as a control. A gene specific primer was used for first strand synthesis, sequence is as follows: FTZ-GSP 5’- GTA ATC TGG AAC ATC GTA TGG GTA -3’.

For the FLAG-TAP RNA-IP experiment, ~ 6.75 x 10^6^ to 1 x 10^7^ U2OS cells were co-transfected with 4.5 μg each of *c-ftz-Δi* +/- *V5-His* and FLAG-TAP plasmids (inserted in p3xFlag-CMV-10 vector, generous gift from H. Cheng) as previously described [56]. 18 to 24 hours post transfection, cells were trypsinized, pelleted and washed 3X times in ice-cold 1X PBS, lysed in 2 ml of IP buffer (20 mM Tris-HCl, pH8, 137 mM NaCl, 1% NP-40, 2 mM EDTA and *cOmplete* mini-protease inhibitor (Roche)) and gently mixed for 25 minutes at 4 °C to ensure complete lysis. The cell lysis was cleared by centrifugation at 16,100*g* for 10 min and 0.45 ml supernatant was mixed with 50μl ANTI-FLAG M2 Affinity Gel beads (A2220, Sigma Aldrich) pre-washed 3X times with IP wash buffer (same as IP buffer, except that 0.05% NP-40 was used) and incubated for 2 to 2.5 hours at 4 °C. As a control experiment, the same amount of cell lysate was added to the same volume of unconjugated Protein A-Sepharose 4B beads (Invitrogen). Following incubation, the beads were washes 4 – 5X times with IP wash buffer and RNA was isolated using the Trizol precipitation as described before. To synthesize the first strand cDNA, a random hexamer primer (ThermoScientific) was used following the SuperscriptIII RT (Invitrogen) manufacturer’s protocol. qRT-PCR was performed as previously described in [13], using the following primers: FTZ-F’ 5’- GCAGGCTCGACTACTTGGAC -3’, FTZ-R’ 5’-GAAATCGCCGGCTCCATTCG -3’, 7SL-F’ 5’- GTG GCG CGT GCC TGT AGT CC -3’ and 7SL-R’ 5’- GGC AAC CTG GTG GTC CCC CG -3’.

### Bioinformatics analysis of 5’SS motif

Sequences for protein-coding genes, 3’UTRs and intergenic regions were downloaded from the UCSD genome browser using Biomart. 3’exons for protein coding genes, and lists of genes with 3’UTR introns were obtained from Ben Blencowe and Ulrich Braunschweig (University of Toronto). Intronless protein-coding genes were downloaded from the intronless gene database [42]. LncRNAs, including 3’exons were downloaded from the LNCipedia website [43]. eRNAs from the FANTOM5 consortium [44] were obtained from Robin Andersson (University of Copenhagen). Starting with TAR files, intronless eRNAs and lncRNAs were computationally sorted. The frequency and expected frequencies for the 5’SS motif were calculated using a Python script (see S1 File for the source code).

## Supporting Information

S1 Fig The *V5-His* element promotes the nuclear speckle-targeting of *β-globin-Δi* mRNA in transfected cells. U2OS cells were transfected with plasmids containing either *β-globin-Δi* or *β-globin-Δi-V5-His* constructs. 18–24 hrs later cells were fixed and stained for *β-globin* mRNA, SC35 and DNA (by DAPI stain) as in Fig 4A. Each row represents a single field of view. The overlay shows *β-globin* mRNA in red and SC35 in green. Examples of nuclear speckles enriched in *β-globin-V5-His* mRNA are denoted by blue arrowheads. Scale bar = 10μm.
(TIF)

S1 File Python script for the analysis of the presence and expected number of 5’SS motifs. (TXT)
