# Supplementary figures and images for: The Consensus 5' Splice Site Motif Inhibits mRNA Nuclear Export
Source: PLoS One. 2015 Mar 31;10(3):e0122743. doi: 10.1371/journal.pone.0122743 (PMC4380460; doi:10.1371/journal.pone.0122743)

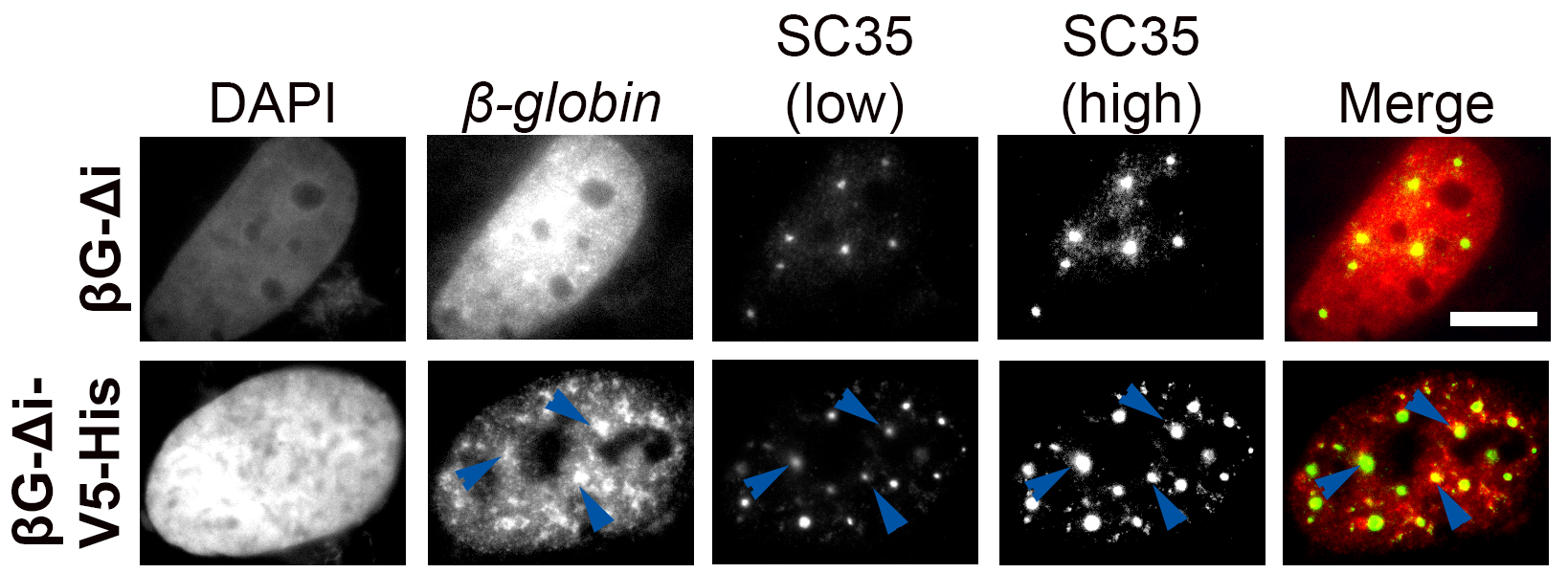

Supplement: S1 Fig — U2OS cells were transfected with plasmids containing either β-globin-Δi or β-globin-Δi-V5-His constructs. 18–24 hrs later cells were fixed and stained for β-globin mRNA, SC35 and DNA (by DAPI stain) as in Fig 4A. Each row represents a single field of view. The overlay shows β-globin mRNA in red and SC35 in green. Examples of nuclear speckles enriched in β-globin-V5-His mRNA are denoted by blue arrowheads. Scale bar = 10μm. (TIF) [file pone.0122743.s001.tif]
